# Supplementary material for: Reassessing chain tilt in the lamellar crystals of polyethylene
Source: Nat Commun. 2023 Sep 21;14:5531. doi: 10.1038/s41467-023-41138-4 (PMC10514264; doi:10.1038/s41467-023-41138-4)
Supplement: Supplementary file 1 — Supplementary Information [file 41467_2023_41138_MOESM1_ESM.pdf]

Supplementary information

Reassessing chain tilt in the lamellar crystals of polyethylene

Shusuke Kanomi[1,2], Hironori Marubayashi[3], Tomohiro Miyata[3] & Hiroshi Jinnai[3,*]

[1]Department of Applied Chemistry, School of Engineering, Tohoku University, 6-6 Aramaki Aza Aoba, Aoba-ku, Sendai, Miyagi 980-8579, Japan

[2]Science & Innovation Center, Mitsubishi Chemical Corporation, 1000 Kamoshida-cho, Aoba-ku, Yokohama, Kanagawa 227-8502, Japan

[3]Institute of Multidisciplinary Research for Advanced Materials, Tohoku University, 2-1-1 Katahira, Aoba-ku, Sendai, Miyagi 980-8577, Japan

*e-mail: hiroshi.jinnai.d4@tohoku.ac.jp

Apex-angle method for evaluating chain tilt angles in lamellar crystals

The ‘apex-angle’ method was previously developed for determining the chain tilt angles (φ) in bulk specimens (Supplementary Figs. 2c and d)[1–4]. In these studies, ultrathin specimens for transmission electron microscopy (TEM) observation were prepared to ensure that the chain axis (*c*-axis) was normal to the electron beam; this was confirmed by examining the morphologies of spherulites and lamellar crystals (for example, via TEM observations of stained and etched specimens) and the average molecular chain orientation (for example, via X-ray diffractometry). Then, the apex angle (γ) of a bent part in lamellar crystals was measured, while the chain direction was roughly known (parallel to green lines in Supplementary Figs. 2c and d). Finally, φ was evaluated by dividing the apex angle with the chain axis. Supplementary Fig. 2c shows a connection of two {101} facets, where $\gamma = 72 + 72 = 144°$ and $\varphi = 90 - 72 = 18°$. Supplementary Fig. 2d shows a connection of {101} and {201} facets, where $\gamma = 72 + 56 = 128°$, $\varphi = 90 - 72 = 18°$, and $90 - 56 = 34°$.

Comparison of WAXD, SAXS, and ED

The averaged (global) structure of polyethylene (PE) was analysed via wide-angle X-ray diffraction (WAXD), small-angle X-ray scattering (SAXS), and microbeam electron

diffraction (ED). Supplementary Fig. 3a shows the WAXD pattern of PE, in which the diffraction rings of {*hk*0}—including the 110, 200, and 020 reflections of orthorhombic PE—are clearly observed. This result unambiguously indicates the random orientation of crystallites within the irradiation area (2×10^5 μm^2). The crystallinity of PE was calculated to be 58.9% using the peak separation method.

Supplementary Fig. 3b shows the SAXS pattern of PE. A ring pattern implies a random orientation of lamellar stacks comprising the lamellar crystals and amorphous layers within the irradiation area (1×10^5 μm^2). The long period (L_p), lamellar thickness (l_c), and amorphous layer thickness (l_a) were calculated to be 30.3, 17.8, and 12.5 nm, respectively, where $L_p = l_c + l_a$. Notably, the WAXD crystallinity exceeded 50%; hence, longer and shorter spacings were assigned to l_c and l_a, respectively, in the SAXS correlation function analysis.

Fig. 2h shows the microbeam ED pattern of PE acquired under moderate conditions [sample temperature: –175 °C, dose rate: 0.1 e$^-$/(Å^2 s), total dose: 0.25 e$^-$/Å^2]. Although the electron-beam area on the sample (~3 μm^2) was several tens of thousands of times smaller than the X-ray beam area ($1–2 \times 10^5$ μm^2), the ED exhibited a ring-like pattern similar to the WAXD pattern.

Although these methods provided statistically averaged information, local structural

information was lost by averaging.

Image reconstruction

In this analysis, information on the diffraction disc intensity and azimuth was extracted by performing image processing on each ED pattern. For more detailed analysis of the crystal orientation and overlapping of multiple crystallites, we plan to incorporate methods such as clustering and pattern matching in the future.

1. A circularly averaged profile (blue line in Supplementary Fig. 5a) was created for the average pattern of all ED patterns acquired via nanodiffraction imaging (NDI). The intensity of the profile decreased with an increase in the scattering angle, and diffraction peaks appeared at approximately 1.6–2.7 nm^{-1}. A background (BG) function was created by fitting two exponential functions [$f(q) = a\mathrm{e}^{-q/b}$] to the region excluding these peaks (the black dashed line in Supplementary Fig. 5a).
2. A BG pattern (Supplementary Fig. 5b) was created using the BG function setup in 1 and subtracted from the raw ED pattern (Supplementary Fig. 5c) to create the pattern shown in Supplementary Fig. 5d. In the raw ED pattern, the intensities around the centre were almost identical to those of the diffraction disc owing to BG and noise. In comparison, in the BG-subtracted pattern, the intensity ratio of the diffraction disc

to other parts was higher.

3. The diffraction discs observed in the ED pattern often did not have the same intensity of pairs symmetric with respect to the centre and did not necessarily exhibit the diameter expected from the convergence angle. The first reason for this is that the incident electron beam deviated slightly from the ideal incident direction. The second reason is the presence of an intensity distribution inside the diffraction disc. Considering the second reason, in contrast to X-ray and neutron diffraction, in which single scattering (kinematic diffraction) is almost established, electron diffraction is considerably affected by multiple scattering (dynamical diffraction). Furthermore, the ED pattern obtained by the focused electron beam in this analysis corresponded to observing the diffraction intensity with slightly different incident angles simultaneously. Therefore, the intensity distribution inside the disc due to these effects contained crucial crystallographic information; however, an ED pattern suitable for such detailed analysis could not be obtained in these measurements. Alternatively, as a process suitable for polymer specimens, we calculated the intensity and azimuth as a pair instead of a single diffraction disk. Supplementary Fig. 5e shows results that are the sum of those in Supplementary Fig. 5d and those rotated 180° with respect to the centre. This process recovered the disc shape,

including weak diffraction points, and the error in the azimuth values was reduced to ~1°.

4. Finally, the processes for removing the slight noise and extracting only the diffraction disk were performed. Supplementary Figs. 5f and g were created using Gaussian filters with two different standard deviations (σ = 2 and 15 pixels; disc diameter = 10 pixels) from Supplementary Fig. 5e, respectively. Then, Supplementary Fig. 5h was created by determining the ratio of the two sets of results (Supplementary Fig. 5f / Supplementary Fig. 5g). Pseudo-peaks appearing at the outer edges of the image were excluded during the image reconstruction stage.
5. Steps 2–4 were performed for all 600 × 600 ED patterns.

Rotation of masks and azimuthal profile creation

A schematic of profile creation is shown in Supplementary Fig. 5i. Azimuth profiles were created from the raw ED pattern (dotted lines in Supplementary Fig. 5j) and the processed pattern (solid lines in Supplementary Fig. 5j). For the raw-pattern profiles, the intensity of the 110-region profile (blue) was always higher than that of the 200-region profile (orange) owing to the inelastic scattering BG. A comparison of two sets of profiles revealed that the processed-pattern profiles had sharper peaks at β = 25°, 87°, and 155°.

This allowed automatic determination of the peak top and calculation of the azimuth even for spots with low intensities. Furthermore, the intensity of the blue profile was higher than that of the orange profile at β = 25° and 87°, with the opposite trend occurring at β = 155°. Thus, the aforementioned process separated the 110 and 200 reflection intensities through a hexagonal-like pattern.

For each scanning position (x, y), azimuthal profiles were created as described in the previous section. The maximum intensities of the 200 reflection profiles were calculated and plotted to obtain the reconstructed dark-field scanning TEM (DF-STEM) image shown in Fig. 3a.

Relationship between electron-beam direction and *c*-axis

The relationship between the electron-beam direction and *c*-axis was established using the following four ED pattern rules:

(i) When a hexagonal-like ED pattern was obtained (for example, Fig. 2e), the electron beam was parallel to the *c*-axis. (The *c*-axis was oriented perpendicular to the plane of the image.)

(ii) When 002 spots were observed, the electron beam was perpendicular to the *c*-axis.

(iii) When two *hk*0 spots were observed, the angle between the electron beam and the *c*-axis (molecular chain) could not be determined.

(iv) When the flat faces of the lamellar crystals were {*h*0*l*} planes[1–4], the lamellae viewed along the *b*-axis appeared to be edge-on. In this case, the *c*-axis was perpendicular to the electron beam (and parallel to the plane of the image), and a pair of 200 spots were observed in the ED patterns (Supplementary Fig. 8).

When the lamellar crystals were viewed edge-on, an image of the lamellae with clear contrast was acquired. However, as the lamellae inclined away from the observing (electron-beam) direction, the apparent lamellar thickness (l_c') increased, and the contrast became ambiguous. The relationship between the tilting angle of the lamellae (ψ) and l_c' is shown in Supplementary Fig. 7, which was constructed using the lamellar thickness estimated via SAXS (l_c = 18 nm). When ψ reached 7°, l_c' was equal to the long spacing value (30.3 nm; l_c + amorphous layer thickness), and the lamellar and amorphous domains merged into a single region. Therefore, when the ~18-nm-thick lamellae were visualised edge-on (revealing clear boundaries between the lamellar and amorphous domains), the tilting angles of the lamellae against the electron-beam direction were <7°. Additionally,

when the lamellae reconstructed from the 200 spots were visualised edge-on, the electron beam was almost perpendicular to the *c*-axis (90 ± ~7°).

Evaluating distribution of tilting angles

1. The lamellar crystals that could be separated from the amorphous and adjacent lamellar crystals were selected from the reconstructed image, and their azimuth angles were measured as straight lines. The lamellar crystals with bends or cranks were divided at the bends to make them straight.
2. The ED pattern of the target area in the lamellar crystal was extracted, and an azimuth profile was created by rotating the circular masks at the scattering angle of the 200 reflection (the pair of orange masks shown in Supplementary Fig. 5i). The azimuth exhibiting the maximum intensity in this profile (orange solid line in Supplementary Fig. 5j) was treated as that of the diffraction spot.
3. Finally, the difference between the azimuth angle (of the normal) of the lamellar crystal and that of the spot (the chain oriented along the normal to the line connecting the spots) in the ED pattern was calculated and used as a histogram.

Notably, dozens of ED spot angles existed for each azimuth angle of a part of the

lamellar crystal. The results for multiple lamellar crystals were summarised in one histogram.

Two types of errors could occur in the measurement of the azimuth angles: (1) the error in measuring the azimuth angles of lamellar crystals and (2) that in measuring the azimuth angles of ED spots automatically. The results were eliminated when the error was (1) several degrees and (2) significantly large. However, not all ED patterns were checked; therefore, the deviation could have been >5° in certain cases.

Supplementary figures

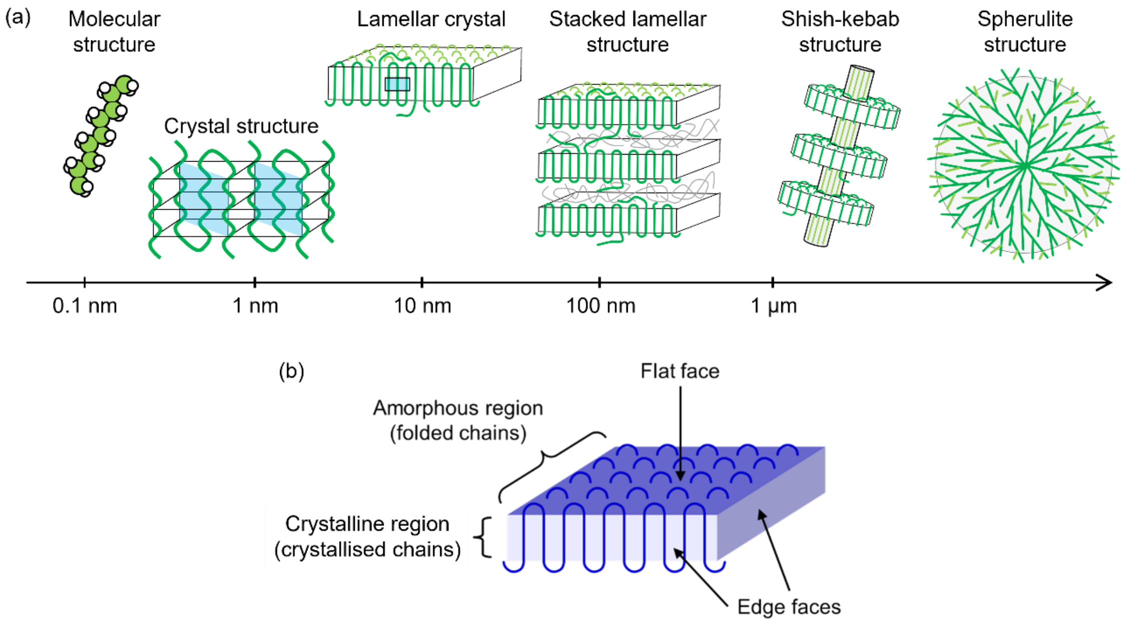

Supplementary Fig. 1 | Hierarchical structure of semicrystalline polymer. a, Schematic showing the hierarchical structures of semicrystalline polymers. **b**, Definition of flat and edge faces in lamellar crystals.

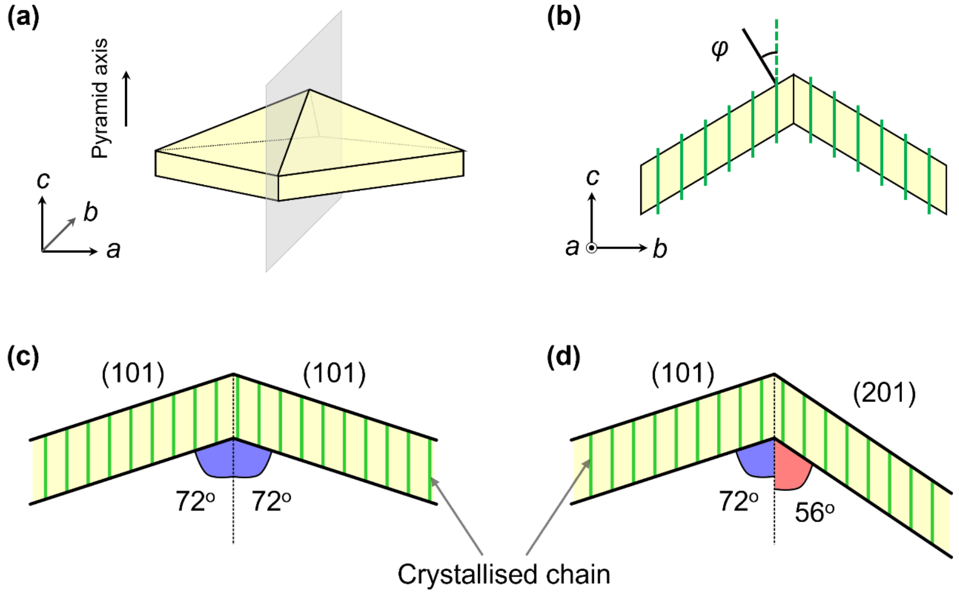

Supplementary Fig. 2 | Schematic of conventional analysis of chain tilt. a,**b**, Schematics of a PE single crystal with a hollow pyramidal shape: **a**, overall view and **b**, *bc* cross-sectional view (grey plane in [**a**]). **c**,**d**, Relationship between intersecting facets and apex angles in the lamellar crystals of bulk PE[11]: connections between lamellae with the **c**, {101} facets and **d**, with the {101} and {201} facets. The angles of 72° and 56° correspond to chain tilt angles of 18° and 34°, respectively.

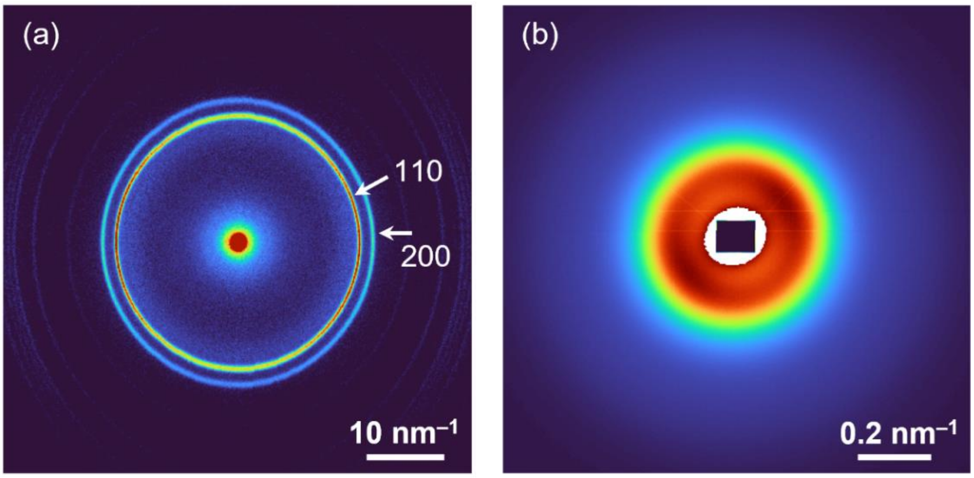

Supplementary Fig. 3 | X-ray diffraction/scattering. a, WAXD and **b**, SAXS patterns of bulk PE. Scale bars denote constant values of q.

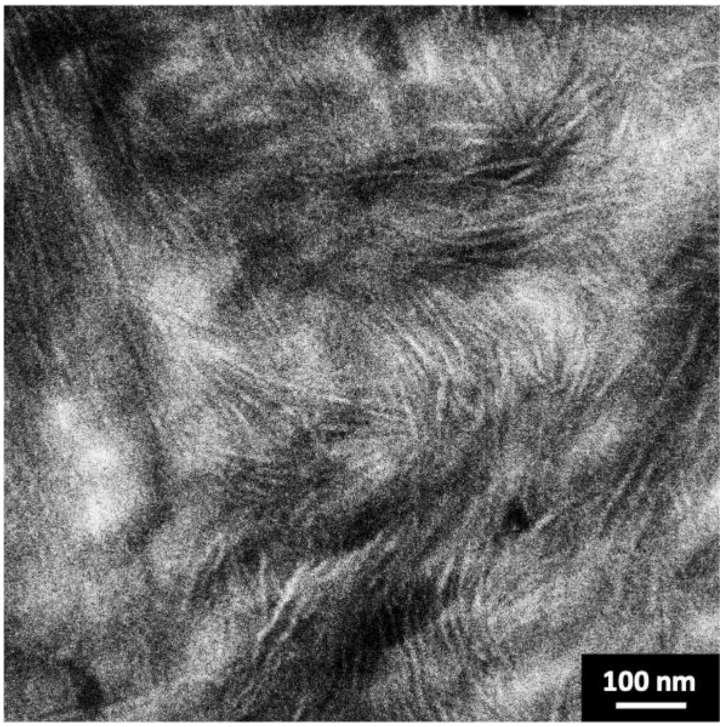

Supplementary Fig. 4 | Staining TEM observation. BF-TEM image of PE stained with RuO_4 (300 Pa, 2 h). Bright and dark regions are crystalline- and amorphous-rich, respectively.

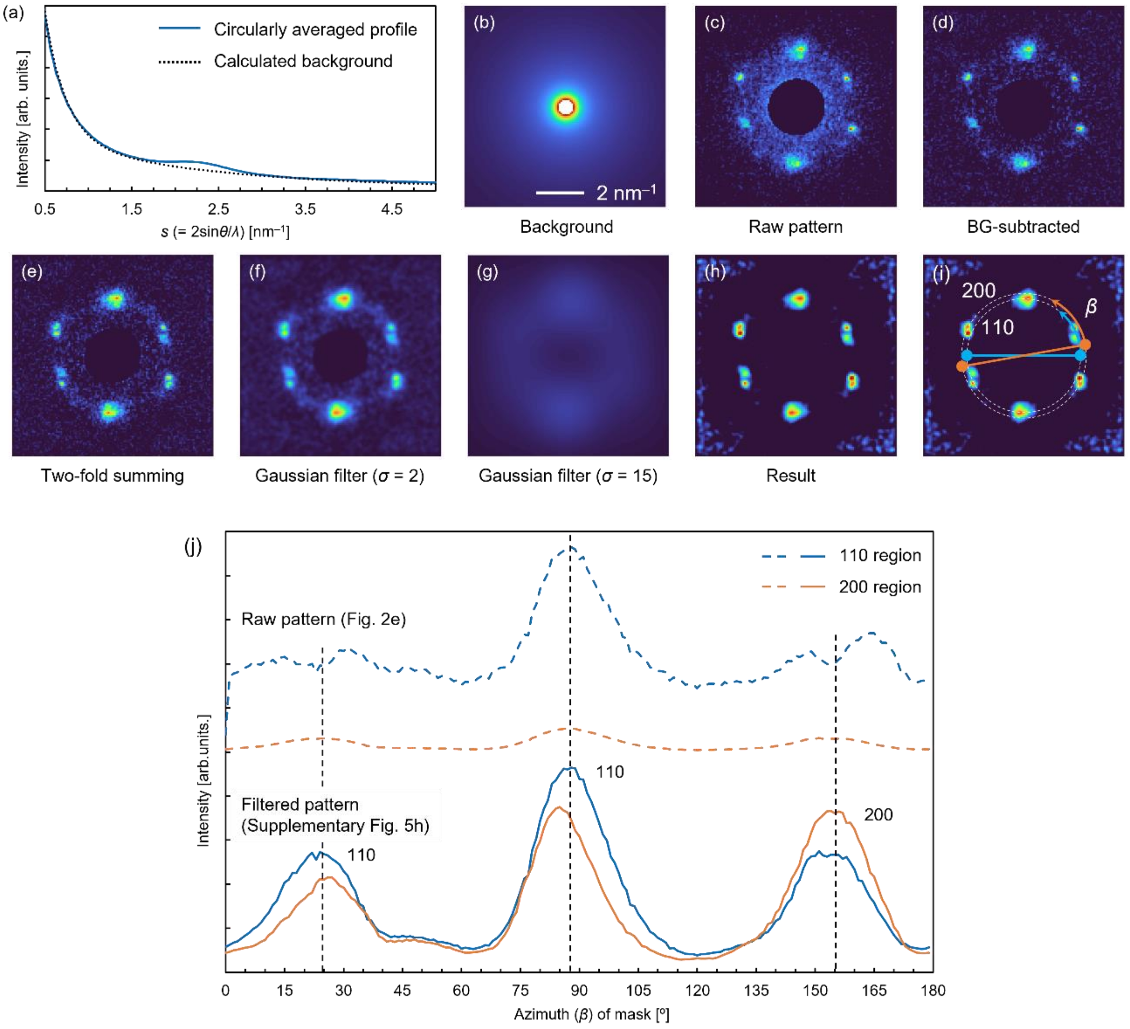

Supplementary Fig. 5 | Image processing. a, Circularly averaged profile of the averaged ED pattern (as in Fig. 2g). The black dashed line indicates the BG created using two exponential functions. **b,** Calculated BG pattern. **c,** Raw ED pattern (Fig. 2e). **d,** BG-subtracted pattern ([c] – [b]). **e,** Two-fold summing pattern, which is the sum of (d) and that rotated by 180° with respect to the centre. **f,g,** Gaussian-filtered patterns with standard deviations of 2 and 15 pixels. **h,** Pattern that is the quotient of the two Gaussian-filtered patterns ([f] / [g]). **i**, Schematic describing the calculation of azimuthal profiles.

Two pairs of circular masks that were symmetric about the centre were created to include the 110 (blue) and 200 (orange) spots and azimuthally scanned. The azimuth angle (β) is defined according to the right-horizontal direction with an anticlockwise rotation being the origin. **j**, Azimuth profiles created by calculating the total intensity inside the two masks per pair at each β.

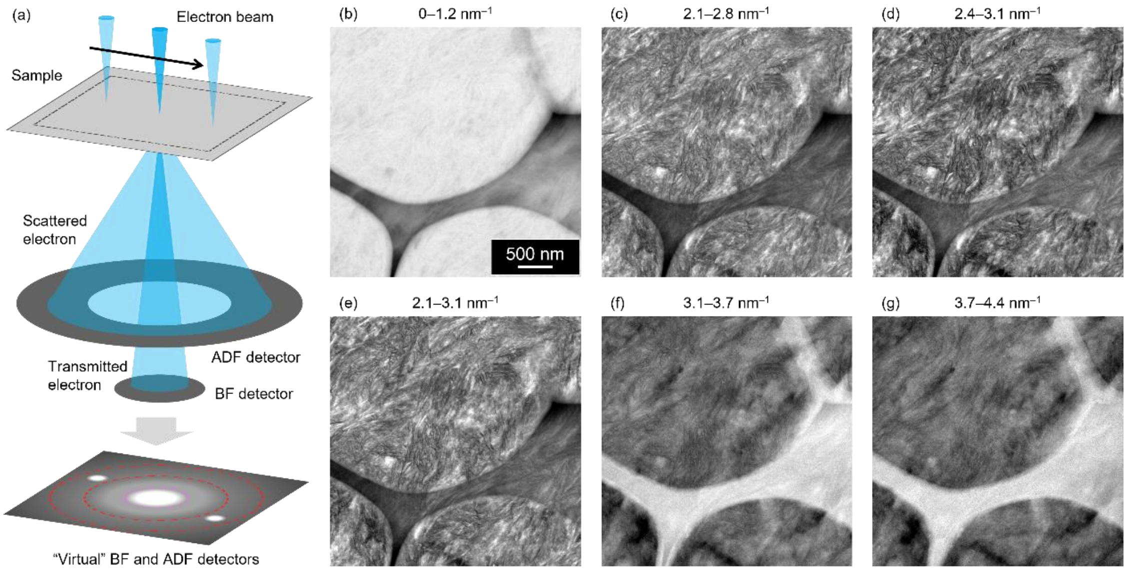

Supplementary Fig. 6 | Reconstructed BF- and ADF-STEM images. a, Arrangement of BF and ADF detectors for STEM, which integrate the intensities of transmitted and scattered electrons, respectively, at each scanning point. The pixelated detector used in NDI recorded ED patterns. A virtual BF or ADF detector was used to reconstruct the STEM images. **b**, Virtual BF-STEM image created using a circular mask with a radius of 1.2 nm^{-1}. **c**–**g**, Virtual ADF-STEM images created using an annular mask. The inner and outer radii of the mask are shown at the top of each image.

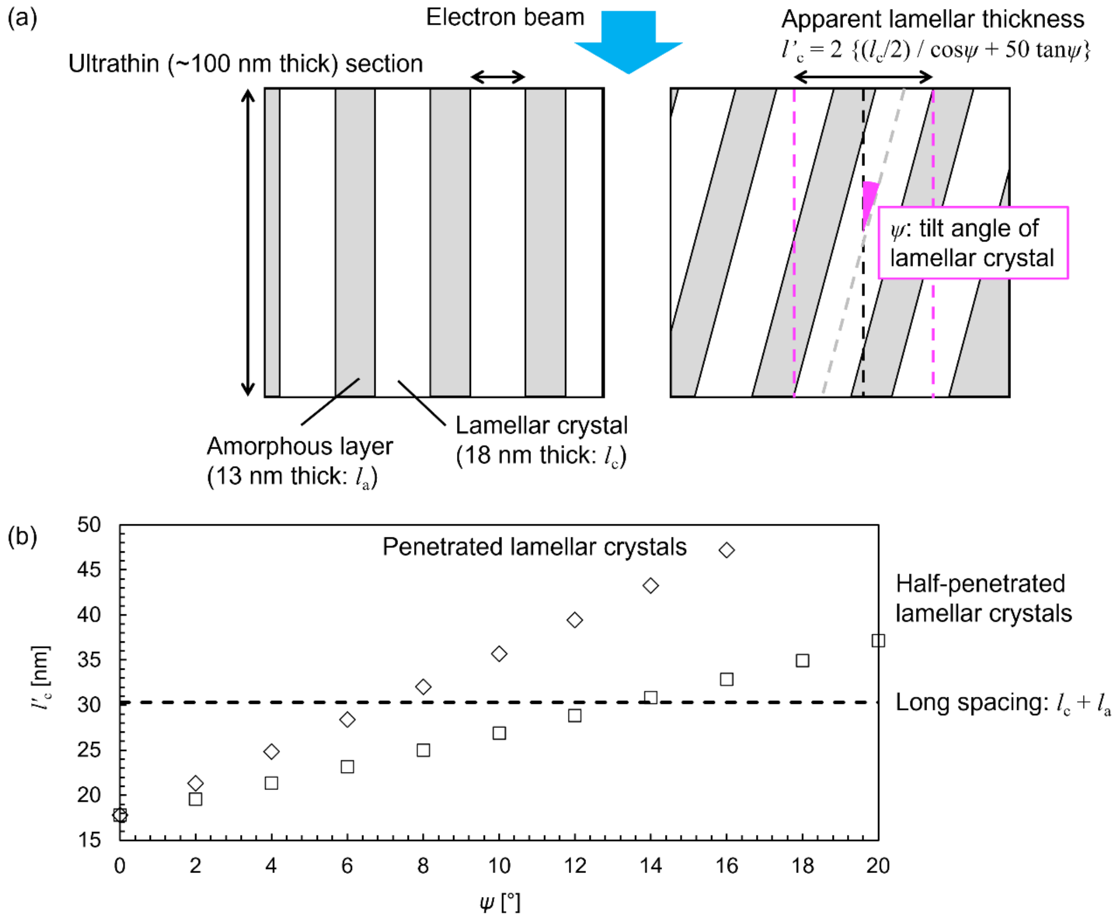

Supplementary Fig. 7 | Crystal/amorphous contrast depending on lamella tilt. a, Schematic depicting the increase in the apparent lamellar thickness (l_c') due to the tilt of the lamellar crystals. **b**, Relationship between the tilt angle of the lamellar crystals (ψ) and l_c'. The dotted line indicates the long spacing (30.3 nm; lamellar thickness + amorphous layer thickness); lamellar crystals were not observed in the reconstructed image when l_c' was higher than this value.

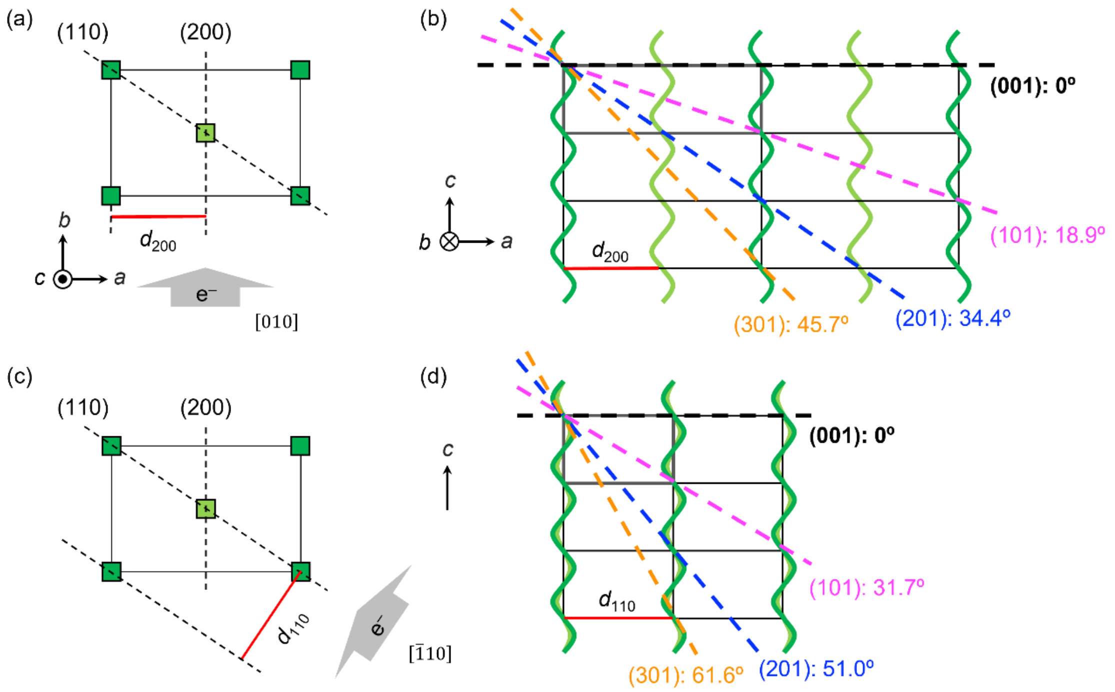

Supplementary Fig. 8 | Schematic of the relationship between the direction of incident electrons and the chain tilt angle φ. Light-green and dark-green objects (filled squares and wavy lines) represent the two PE chains packed in the orthorhombic unit cell with different setting angles. (**a**) and (**c**) show the crystallographic unit cells viewed from [001] (the *c*-axis projection). The direction of incident electrons is indicated by grey arrows. (**b**) shows the unit cells viewed from [010] (the *b*-axis projection) and corresponds to (**a**). (**d**) shows the unit cells viewed from $[\bar{1}10]$ and corresponds to (**c**). **b**,**d**, Miller indices (*hkl*) of the flat faces of lamellar crystals and the corresponding φ values.

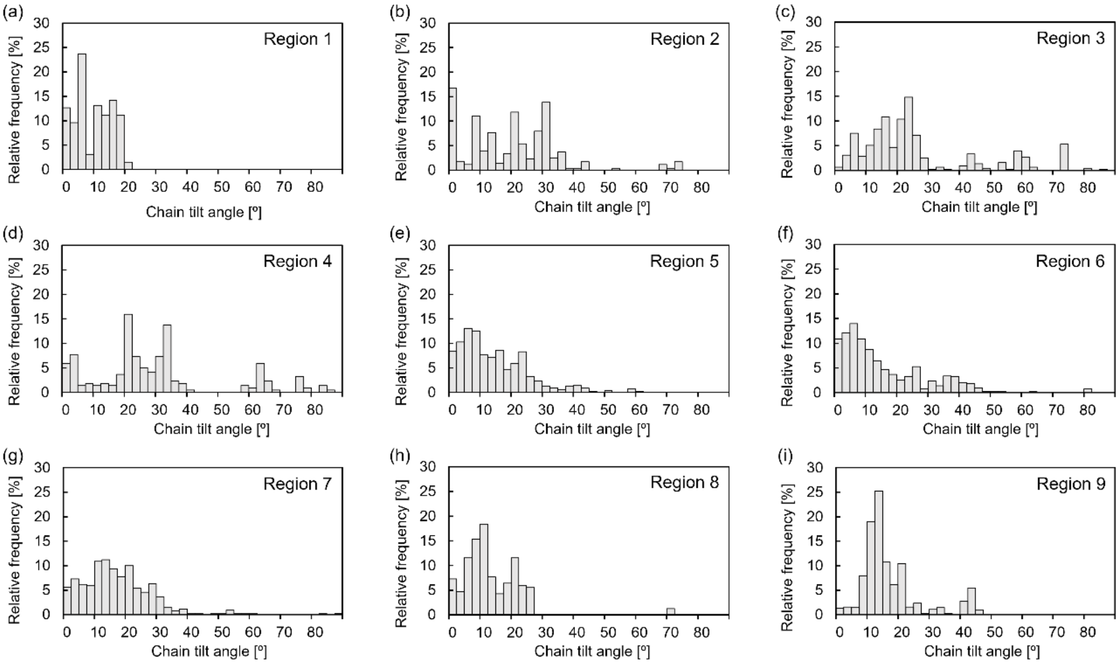

Supplementary Fig. 9 | Histograms of chain tilt angles. a–i, Histograms of φ obtained from Regions 1–9 (red frames) in Fig. 3a, respectively.

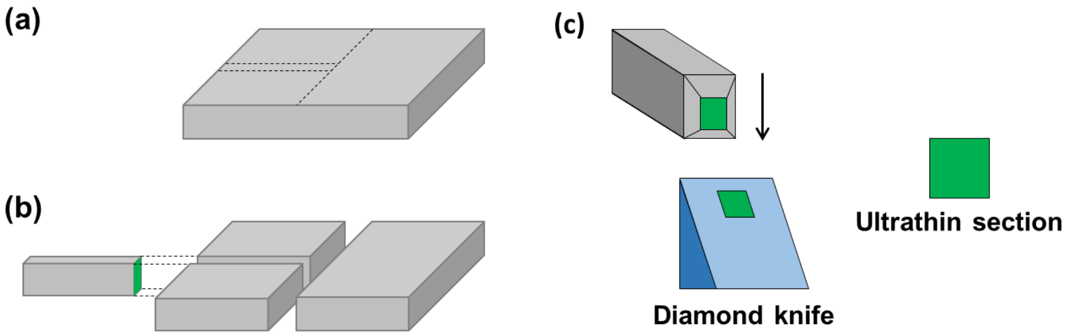

Supplementary Fig. 10 | Schematic describing the preparation of ultrathin sections.

The central part (**b**) of a crystallised film (**a**) was cut off, and ultrathin sections parallel to the sides of the specimen were prepared (**c**).

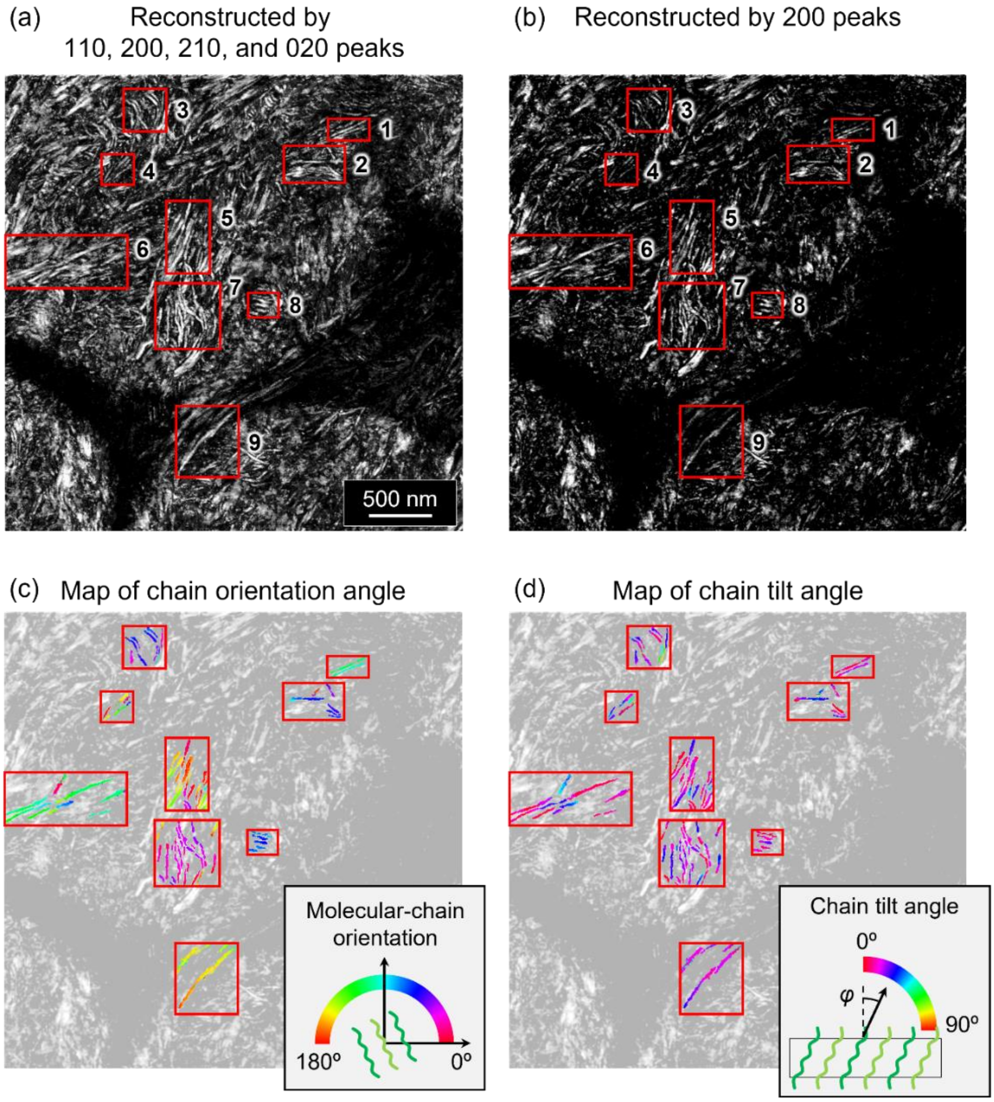

Supplementary Fig. 11 | Reconstructed image and maps of area 1. a, DF-STEM image of HDPE reconstructed by summing the intensities of the 110, 200, 210, and 020 peaks. This image was created to visualise as many edge-on lamellae as possible. **b**, DF-STEM image of HDPE reconstructed by the 200 peak intensity (Fig. 3a with different contrast). **c**, Map of the chain orientation angles in the Cartesian coordinate system (absolute molecular chain orientations, as in Fig. 3b). **d**, Map of the chain tilt angle φ (as in Fig. 4a).

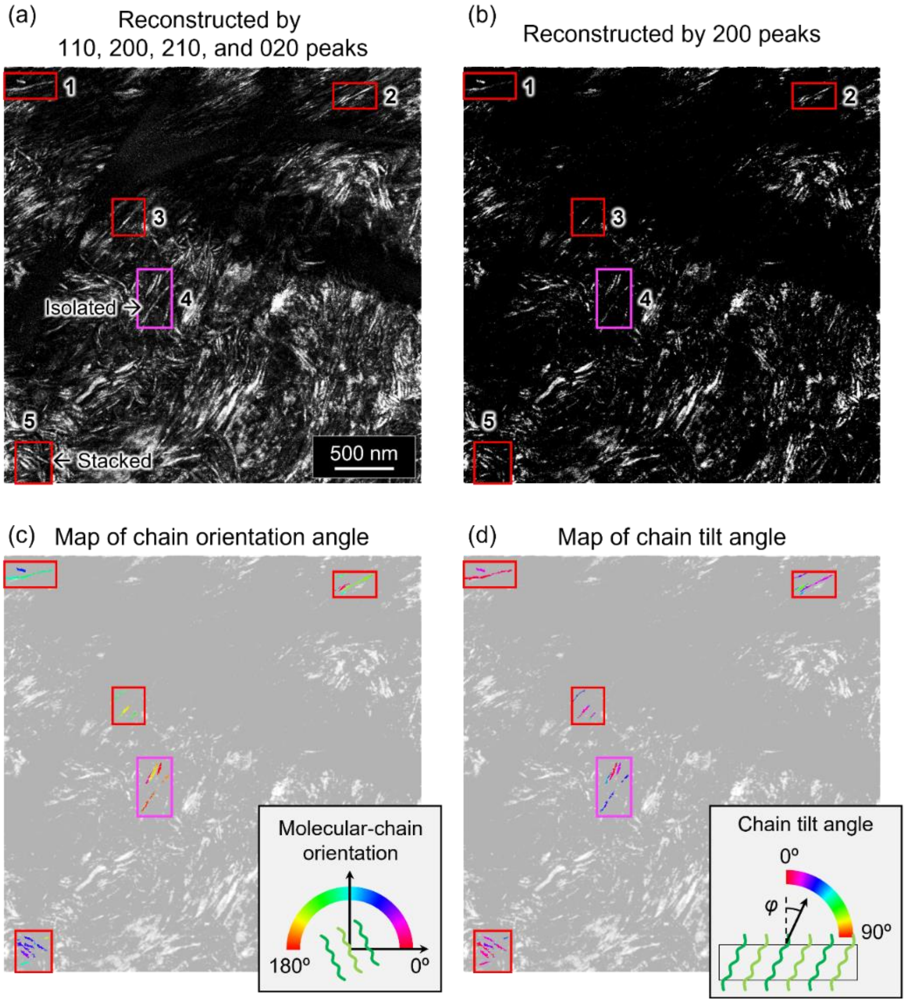

Supplementary Fig. 12 | Reconstructed image and maps of area 2. a, DF-STEM image of HDPE reconstructed by summing the intensities of the 110, 200, 210, and 020 peaks. Note that this image has a different field of view (in the same ultrathin section) from the images in the main-text figures and Supplementary Figs. 11 and 13. The electron-beam scanning was performed at 5-nm intervals (6-nm intervals were used for Figs. 3 and 4). **b**, DF-STEM image of HDPE reconstructed by the 200 peak intensity. **c**, Map of the chain orientation angles in the Cartesian coordinate system (absolute molecular chain orientations). **d**, Map of the chain tilt angle φ.

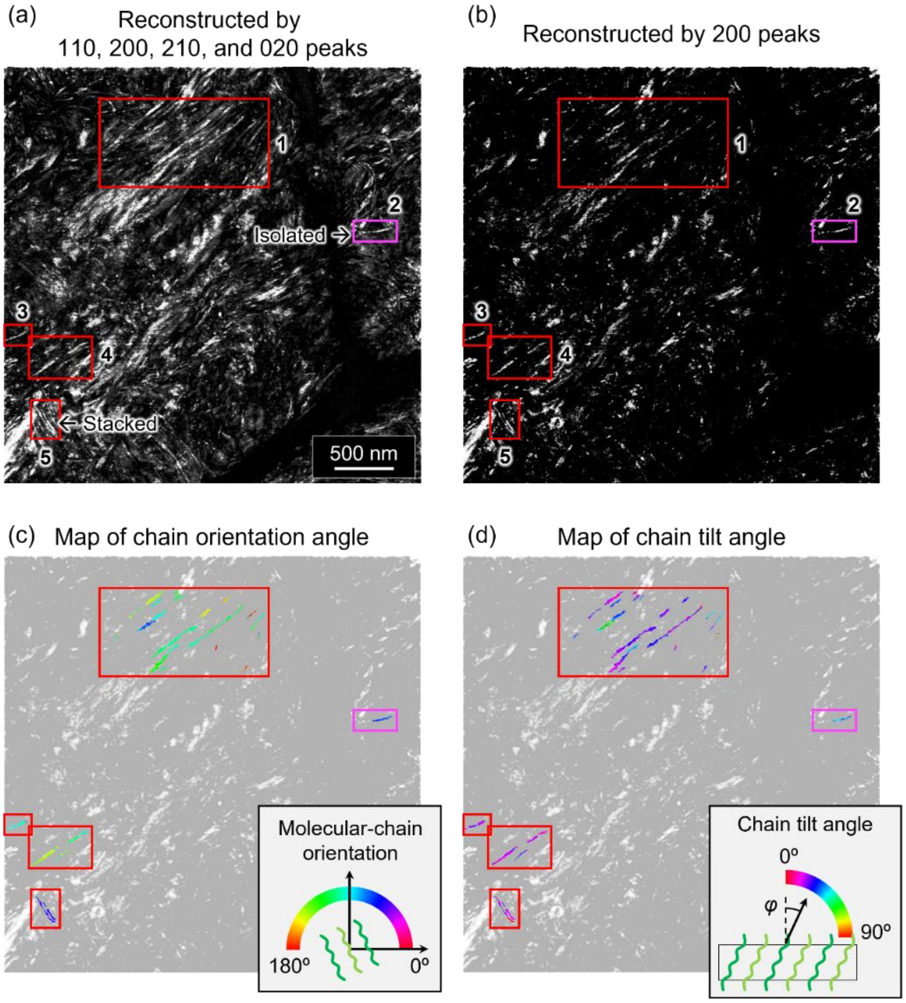

Supplementary Fig. 13 | Reconstructed image and maps of area 3. a, DF-STEM image of HDPE reconstructed by summing the intensities of the 110, 200, 210, and 020 peaks. This image has a different field of view (in the same ultrathin section) from the images in the main-text figures and Supplementary Figs. 11 and 12. The electron-beam scanning was performed at 5-nm intervals (6-nm intervals were used for Figs. 3 and 4). **b**, DF-STEM image of HDPE reconstructed by the 200 peak intensity. **c**, Map of the chain orientation angles in the Cartesian coordinate system (absolute molecular chain orientations). **d**, Map of the chain tilt angle φ.

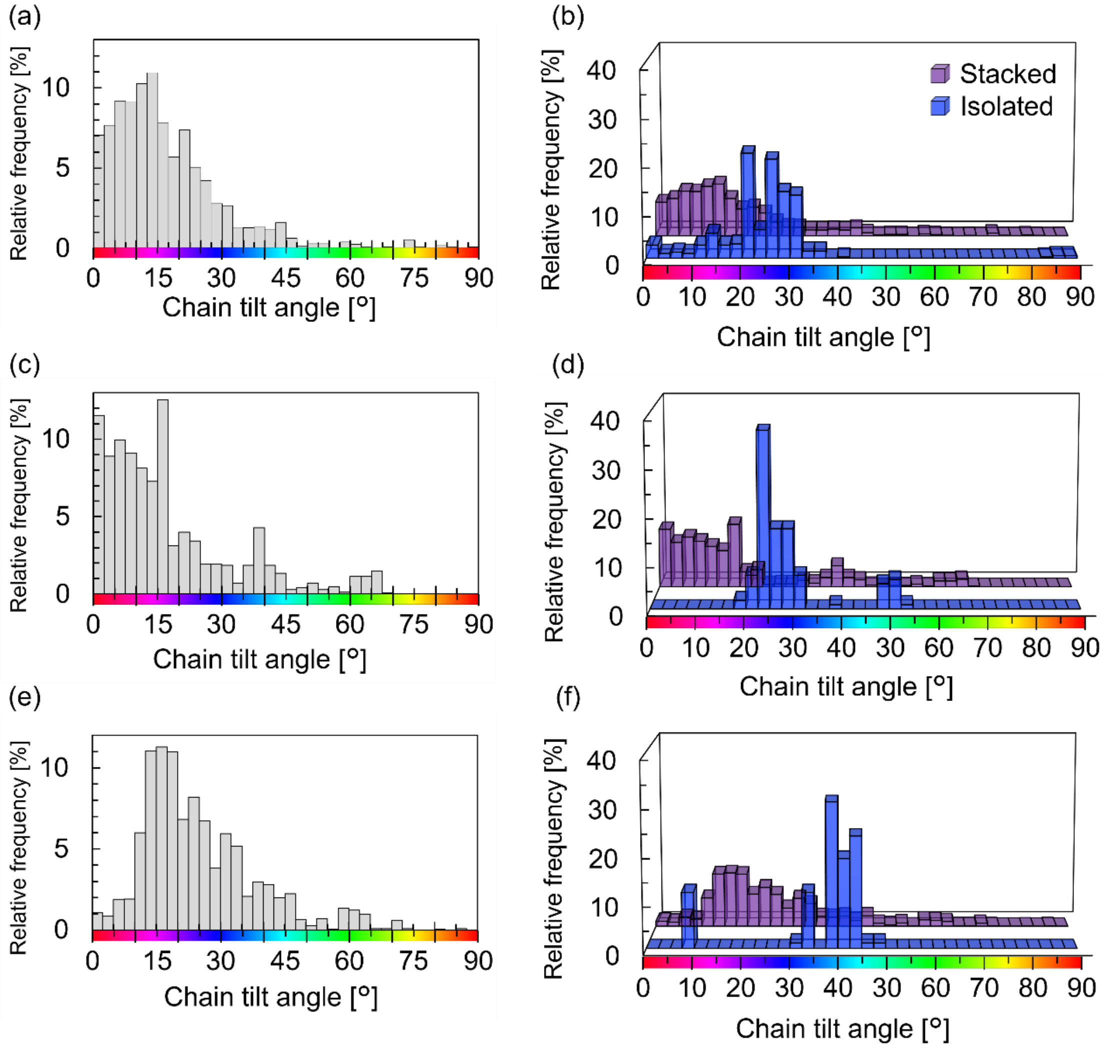

Supplementary Fig. 14 | Summary of histogram from area 1-3. a,c,e, Histograms of φ for the lamellae shown in Supplementary Figs. 11, 12, and 13, respectively. **b,d,f,** Histograms of φ for isolated and stacked lamellae shown in Supplementary Figs. 11, 12, and 13, respectively. Because stacked lamellae were more prevalent in the present specimen, the integrated histograms shown in a, c, and e exhibited similar features to the histograms for the stacked lamellae.

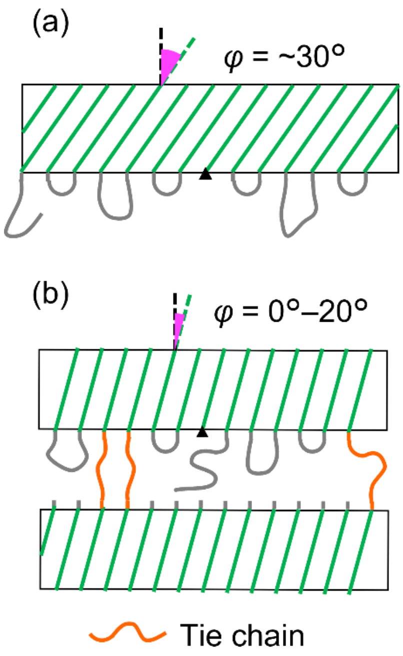

Supplementary Fig. 15 | Speculated structure model of isolated and stacked lamellae.

a,**b**, Structure model of **a**, isolated and **b**, stacked lamellae. Chains in the lamellar crystal and amorphous region are shown as green and grey lines. Tie chains that bridge two or more neighbouring lamellae are shown as orange lines.

References

1. Bassett, D. C. & Hodge, A. M. On lamellar organization in certain polyethylene spherulites. *Proc. R. Soc. A* **359,** 121–132 (1978).
2. Bassett, D. C. & Hodge, A. M. On the morphology of melt-crystallized polyethylene - I. Lamellar profiles. *Proc. R. Soc. A* **377,** 25–37 (1981).
3. Voigt-Martin, I. G. & Mandelkern, L. A quantitative electron-microscopic study of a linear polyethylene fraction crystallized at different temperatures. *J. Polym. Sci. Polym. Phys. Ed.* **19,** 1769–1790 (1981).
4. Stack, G. M., Mandelkern, L. & Voigt-Martin, I. G. Crystallization, melting, and morphology of low molecular weight polyethylene fractions. *Macromolecules* **17,** 321–331 (1984).
